# Fatal influenza A (H5N1) virus Infection in zoo-housed Tigers in Yunnan Province, China

**DOI:** 10.1038/srep25845

**Published:** 2016-05-10

**Authors:** Tingsong Hu, Huanyun Zhao, Yan Zhang, Wendong Zhang, Qiang Kong, Zhixiao Zhang, Qinghua Cui, Wei Qiu, Bo Deng, Quanshui Fan, Fuqiang Zhang

**Affiliations:** 1 Centre for Disease Control and Prevention, Chengdu Military Region, Kunming 650118, China; 2 Centre for Animal Disease Control and Prevention, Yunnan Province, Kunming 650051, China; 3 Department of Biochemistry and Molecular Biology, Fudan University Shanghai Medical College, Shanghai 200030, China; 4 Yunnan Agriculture University, Kunming 650223, China

## Abstract

From 2014 to 2015, three cases of highly pathogenic avian influenza infection occurred in zoo-housed north-east China tigers (*Panthera tigris ssp.altaica*) and four tigers died of respiratory distress in succession in Yunnan Province, China. We isolated and characterized three highly pathogenic avian influenza A(H5N1) viruses from these tigers. Phylogenetic analysis indicated that A/tiger /Yunnan /tig1404 /2014(H5N1) belongs to the provisional subclade 2.3.4.4e which were novel reassortant influenza A (H5N1) viruses with six internal genes from avian influenza A (H5N2) viruses. The HA gene of the isolated A/tiger /Yunnan /tig1412 /2014(H5N1) virus belongs to the subclade 2.3.2.1b. The isolated A/tiger /Yunnan /tig1508/2015 (H5N1) virus was a novel reassortant influenza A (H5N1) virus with three internal genes (PB2, PB1 and M) from H9N2 virus and belongs to the subclade 2.3.2.1c.

Our results suggested that the *reassortant* different H5N1 virus sublineages can successfully cross species barriers from avian to mammal and infect north-east China tigers.

Highly pathogenic avian influenza (HPAI) A (H5N1) viruses were first detected in China in 1996 (prototype strain A/goose/Guangdong/1/96 [Gs/GD])^1^ and have since spread causing outbreaks in poultry and wild birds in more than 70 countries^2^,^3^. Recently, HPAI viruses have crossed the species barrier from avian to mammal and caused human and other mammal infections. In 1997, HPAI H5N1 virus caused the first cases of human infection in Hong Kong^4^,^5^, and reemerged in 2003. So far, more than 650 cases of human infections HPAI A H5N1 virus (with around 60% fatality) have been reported in 16 countries since 2003 (WHO. WCnochcoaiAHNrt. http://www.who.int/influenza/ human animal interface /EN GIP 20140124 Cumulative Number H5N1cases.pdf.). HPAI A H5N1 viruses infections have also been reported in the globe amongst cats, tigers, leopards and other felids since 2004^6^.

we have reported the novel clade 2.3.4.4 influenza A (H5N1) virus caused a major wave of highly pathogenic avian influenza outbreak in poultry in the Yunnan Province, China from December 2013 to March 2014^7^. Here, we report three cases fatal influenza A (H5N1) virus infection in zoo-housed Tigers in Yunnan Province, China.

From 2014 to 2015, three cases of highly pathogenic avian influenza infection occurred in zoo-housed north-east China tigers (*Panthera tigris ssp.altaica*) and four tigers died of respiratory distress in Yunnan Province, China. We isolated and characterized three highly pathogenic avian influenza A (H5N1) viruses from tigers. The isolated viruses were named A/tiger /Yunnan /tig1404 /2014(H5N1), A/tiger /Yunnan /tig1412 /2014(H5N1) and A/tiger /Yunnan /tig1508 /2015(H5N1). Full genome influenza sequences and analyses have been performed. Sequence analyses revealed that the three viruses belonged to different clades.

## Results

### Case Descriptions

On April 8^th^, 2014, one 12monthold female tiger died in a zoo. The zookeepers reported that there were a total of fifty tigers in the zoo, and four tigers were reared in one cage. The tigers were fed daily with cooked chicken carcasses, pork and beef from the market. Two days prior to its death, the tiger displayed varying degrees of clinical symptoms, including high fever, vomiting with copious amounts of green-yellow liquid and severe respiratory distress. On December 15^th^, 2014, another 6monthold male tiger died in the zoo with similar clinical symptoms: high fever three days, vomiting and severe respiratory distress. On August 12^th^, 2015, two more tigers died in the zoo; one 8monthold male tiger died from high fever two days following vomiting and severe respiratory distress, and the other 24monthold female tiger was a sudden death. After the first tiger died, the other tigers were fed daily just with pork and beef, no chicken. But five peacocks died with H5N1 virus infection in the zoo, on June.

**Figure 1:**
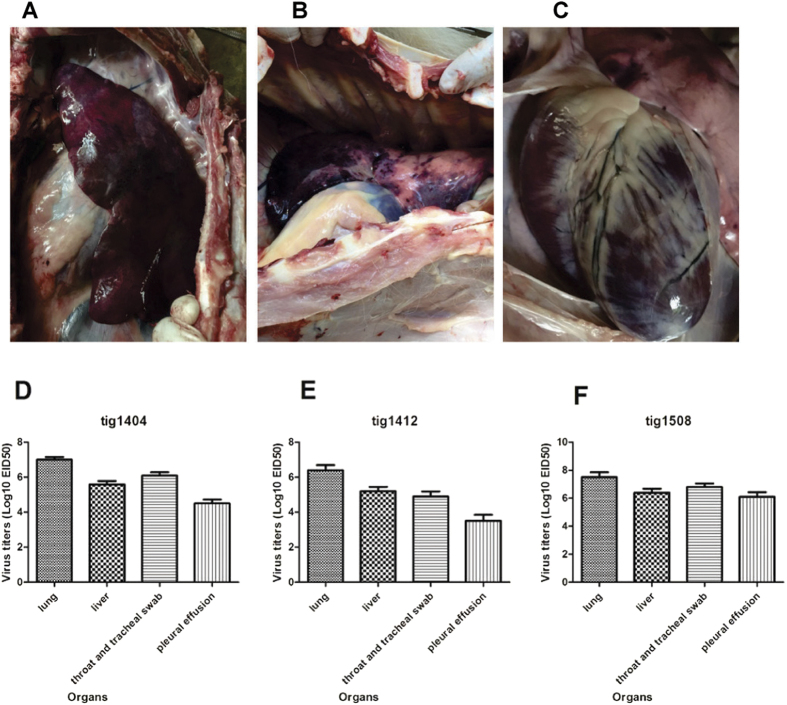
Autopsy change and The H5N1 viral titers in different organs of the tigers. Autopsy change in different organs of tig1508 are shown in (**A**) (liver), (**B**) (lung) and (**C**) (heart); the viral titers in different organs of tigers are shown in (**D**) (tig1404), (**E**) (tig1412) and (**F**) (tig1508).

All tigers that died had nasal discharge and neurologic signs of infection. Necropsy was performed at once after their death. The lungs and livers were severely congested with hemorrhaging (Fig. 1A–C, tig1508), the brains had severe congestion; pleural effusion was observed, and serosanguinous exudate was also seen throughout the tracheal and bronchiolar lumen in all deceased tigers. RNA from the lung, liver, pleural effusion, throat and tracheal swab specimens tested positive for the hemagglutinin gene and neuraminidase gene of the H5N1 virus. The homogenates of the RT-PCR positive samples for tigers were centrifuged at lowspeed and either undiluted or 10-fold serially diluted supernatants, and then inoculated into 10-day-old SPF embryonated chicken eggs. The viral titers in the tigers were calculated using the Reed and Muench method and were highest in the lungs (Fig. 1D,E).

### Virus isolation and genetic identity analysis

**Figure 2:**
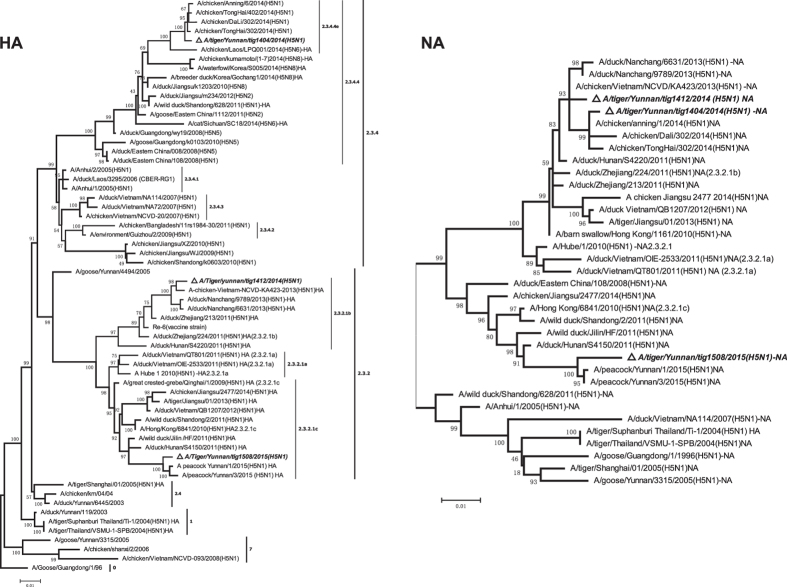
Phylogenetic tree of influenza A (H5N1) hemagglutinin (HA) gene and neuraminidase(NA) gene sequences from the tiger isolates in Yunnan and related reference viruses retrieved from the GenBank database. The phylogenetic tree was generated in MEGA version 6 (www.megasoftware. net), using maximum likelihood (ML) analysis with 1,000 bootstrap replicates. The HA tree was rooted to prototype strain A/goose/Guangdong/1/96 [Gs/GD]. Scale bar indicates nucleotide substitutions per site. The viruses reported in this paper were highlighted using black triangle and bold italic. Note*:some 2.3.4.4 sub-clades such as 2.3.4.4e have just been proposed but not yet been confirmed by the WHO/OIE/FAO H5N1 nomenclature working group.

**Figure 3:**
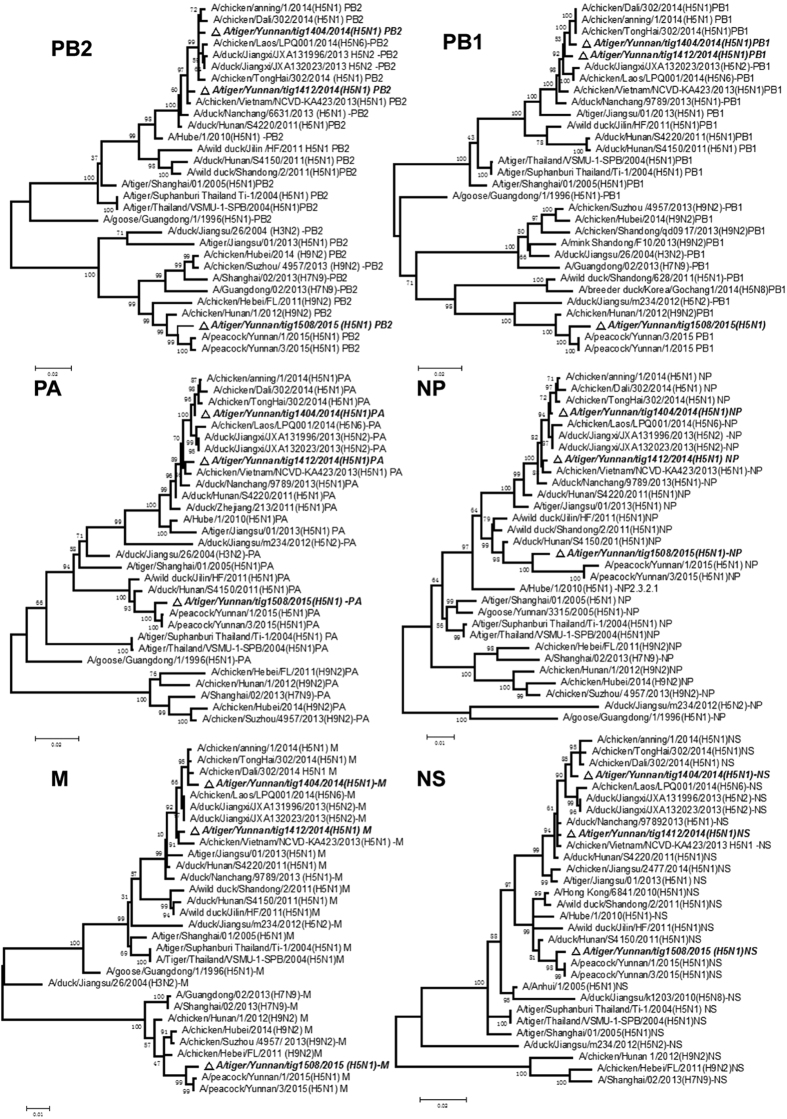
Phylogenetic analyses of six internal genes of the tiger isolates in Yunnan and related reference viruses. The viruses reported in this paper were highlighted using black using black triangle and bold italic. PB2, basic polymerase 2; PB1, basic polymerase 1; PA, acidic polymerase; NP, nucleoprotein; M, matrix protein and NS, nonstructural protein.

To analyze the correlation of H5N1 virus infection in tigers and peacocks in the zoo, two peacock isolates (A/ peacock/Yunnan /1 /2015(H5N1) and A/peacock /Yunnan /3 /2015(H5N1)) and three tiger originated virus isolates (tig1404, tig1412 and tig1508 isolated from tigers died on April 8^th^, December 15^th^, 2014 and August 12^th^(the 8monthold male tiger), 2015, respectively.) were chosen for full genome sequencing. Sequence analyses revealed that the three viruses belonged to different clades. Phylogenetic analysis indicated that HA, NA and six internal genes of A/tiger /Yunnan /tig1404 /2014(H5N1) shared more 99% homology (Table 1) and clustered with A/chicken /Tonghai/ 302 /2014(H5N1), and belongs to provisional subclade 2.3.4.4e (Figs 2 and 3) which were reassortant influenza A (H5N1) viruses with six internal genes originated from avian influenza A (H5N2) virus: A/duck/Jiangxi/JXA132023/(H5N2) in 2013, and associated with the outbreak of H5N1 occurred in chicken in Yunnan Province from December 2013 to March 2014^7^. The eight gene segments of A/tiger /Yunnan /tig1412 /2014(H5N1) were most closely related to A/chicken /Vietnam /NCVD- KA423 /2013 (H5N1) and A/duck /Nanchang /6631 /2013 (H5N1, isolates from China) from the subclade 2.3.2.1b (Figs 2 and 3) with more 99% nucleotide sequence identity (Table 1).

Homological analysis showed that A/tiger /Yunnan /tig1508 /2015(H5N1) and peacock isolates (including A/peacock/Yunnan /1 /2015(H5N1) and A/peacock /Yunnan /3 /2015(H5N1)) shared 99.0%, 98.8%, 98.3%, 98.6%, 99.1%, 97.5%, 99.2% and 99.3% nucleotide identities with HA, NA, PB2, PB1, PA, NP, M and NS genes, respectively. The highest homology of the tig1508 isolate virus genome were as follows (in Table 1): 97.6%, 97.1%, 98.2%, 98.2% and 98.8% homology with the HA, NA, PA, NP and NS genes of A/duck /Hunan /S4150 /2011(H5N1) belonging to Clade 2.3.2.1c, 98.1% and 98.2% with the PB2 and PB1 genes of A/chicken/ Hunan/1/2012(H9N2) and 98.4% with the M gene of A/chicken/ Hebei /FL/ 2011(H9N2). Evolutionary analysis showed that A/tiger /Yunnan /tig1508 /2015(H5N1) including peacock isolates is a novel reassortant virus originating from H5N1 and H9N2 subtypes influenza A virus. The HA, NA and three internal genes (PA, NP, NS) of the novel H5N1 virus originated from A/duck /Hunan /S4150 /2011(H5N1) belonging to subclade 2.3.2.1c and the other three internal genes originated from avian influenza A (H9N2) virus: PB2 and PB1 segments originated from A/chicken/ Hunan/1/2012(H9N2) and M segment originated from A/chicken/ Hebei /FL/ 2011(H9N2) (Figs 2 and 3).

### Molecular features associated with AIV virulence, transmissibility, and antiviral resistance

We analyzed the molecular features of tiger originated viruses associated with H5N1 virus virulence, transmissibility, and antiviral resistance. The three isolates displayed some molecular markers associated with increased virulence and transmission in mammals according to the H5N1 Genetic Changes Inventory (http://www.cdc.gov/ flu/ pdf/ avianflu/ h5n1-inventory.pdf). The isolated hemagglutinin cleavage sites possess a multibasic amino acid cleavage site (Table 2). Although the three isolates possessed a conserved amino acid motif Q-R-G (Gln Ser Gly equivalent to clades 2.3.4 and 2.3.2) at residues 222–224 (H3 numbering 226–228) of the hemagglutinin protein indicating no substantial changes in avian-like receptor binding preferences^8^,^9^, the HA of the viruses isolate from the tigers harbored some mutations (such as Asp94Asn in tig1404 and tig1508, Ser133Ala in tig1404, tig1412 and tig1508, Thr156Ala in tig1412, Thr188Ile in tig1412 and tig1508, and Lys189Arg in tig1412), which several studies have suggested increased the binding of the H5N1 virus to the sialic acid (SA) α-2, 6-Gal (α2-6) receptor^10^,^11^,^12^. The neuraminidase stalk deletion (49–68 deletion) was detected in the three isolates, which could play a role in enhancing H5N1 virulence in mice^13^. The mutations/substitutions involved in drug resistance and transmission to mammals, were observed in the NA (His254Tyr) of the three isolates and PB2 (Asp701Asn) proteins of tig1508 isolate (Table 2)^14^,^15^. The three isolates possessed Asn30Asp and Thr215Ala mutation in M1 protein, which are associated with increased virulence in mice^16^. The Ser31Asn mutation was exhibited in the M2 protein of the tig1508 virus, which confers resistance to the antiviral drug adamantane and rimantadine^17^,^18^. The mutations of NS1 protein plays an important role in enhancing the virulence of H5N1 viruses in mice, such as 80–84 deletion, Leu98Phe, Ile101Met and PDZ ligand motif ESEV in 222–225 sites^19^,^20^, were observed in the three isolates from the tigers and Asp87Glu in the tig1404 and tg1412 isoslates.

The N-Glycosylation sites were predicted to examine the sequence context of Asn-Xaa-Ser/Thr sequins by the NetNglyc server 1.0. In addition to seven possible N-glycosylation sites observed for other serotype H5N1 in hemagglutinin proteins^9^, the predicted results of the N-Glycosylation sites indicated that the HA segments of each isolate harbored new N-glycosylation sites: Asn84 and Asn154 in the tig1404 isolate, Asn273 in the tig1412 isolate, and Asn154 in the tig1508 isolate, respectively.

In summary, all of mutations/substitutions of the gene segments of the tiger originated viruses could contribute to the enhancement of virulence or the increase of the H5N1 virus binding to the α2-6 receptor.

### Pathogenicity of the tigers originated H5N1 viruses in mice

**Figure 4:**
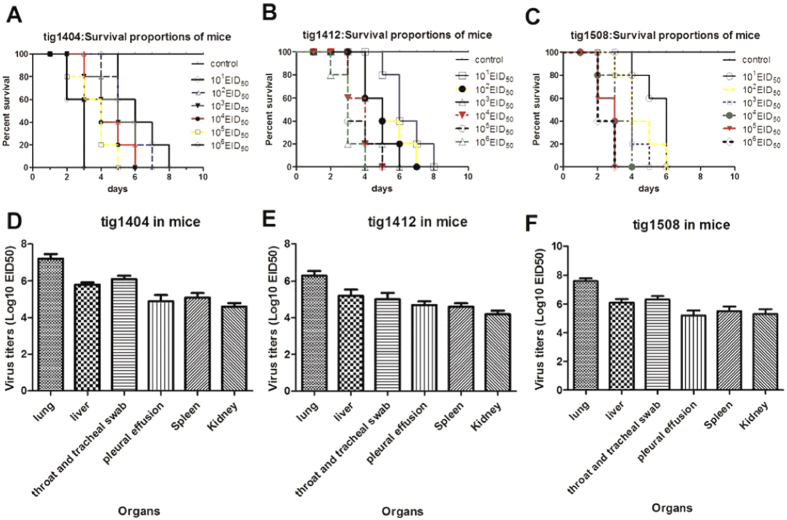
Virulence of tigers-originated- H5N1 viruses in mice. The survival rate of infected mice are shown in (**A**) (tig1404), (**B**) (tig1412) and (**C**) (tig1508); the tigers-originated- H5N1 viruses were detected in lung, liver, pleural effusion, throat and tracheal swab, Kidney and Spleen of infected mice and the viral titers in each organ of mice after challenge with influenza are shown in (**D**) (tig1404), (**E**) (tig1412) and (**F**) (tig1508).

To further characterize the virulence of these novel tigers-originated H5N1 viruses in mammals, we infected mice with these viruses and observed for 10 days for morbidity. The signs of illness, anorexia and dyspnea were observed in the mice inoculated with 10^1^-10^6^ ELD_50_ and the mice inoculated with 10^6.0^ EID_50_ began to die at 2 days post infection (dpi); by 8 dpi, all mice in the experimental groups had died(Fig. 4A–C). Among the mice infected with tig1404, tig1412 and tig1508, viruses were positive with RT-PCR and were successfully re-isolated from lung, liver, pleural effusion, throat and tracheal swab, Kidney and Spleen tissues. Viral titers in eggs were calculated and shown in the Fig. 4 (Fig. 4D–F). Virus replication was not detected in any of the direct contact mice in the tig1404 and tig1412 groups. But in the Tig1508 group, virus replication was detected in one direct contact naïve mouse. These results indicated that these tigers-originated H5N1 viruses caused infection and death in mice and the tig1508 virus maybe have the ability for transmission and infection in mice through direct contact.

### The hemagglutination inhibition (HI) test

The HI titers of Re-5 antiserum against subtype tig1404, tig1412 and tig1508 isolate was 4.825 ± 1.083, 4.289 ± 1.160 and 4.232 ± 1.212 log2, respectively. Re-6 antiserum specimens (n = 38) collected from healthy chickens vaccinated twice with the Re-6 vaccine strain (belonging to clade 2.3.2.1b) (Jan 2014). The HI titers of Re-6 antiserum against subtype tig1404, tig1412 and tig1508 isolate was 4.475 ± 1.753, 6.75 ± 0.840 and 3.675 ± 1.163 log2, respectively(Table 3). If HI titers of the tested serum were higher than 5 log2, they were determined to be positive. As suggested by the HA1 protein sequence differences, clade 2.3.2.1b antiserum (from the RE-6 vaccine strain) inhibited hemagglutination of a representative clade 2.3.2.1b virus including tig1412 isolate, but did not inhibit hemagglutination of clade 2.3.2.1c such as tig1508 isolate, a representative clade 2.3.4 RE-5 (vaccine strain) and tig1404 isolate.

## Discussion

In this study, we provide the evidence of fatal H5N1 AIV infection in zoo-housed Tigers in Yunnan Province. In comparisons of previously published nucleotide sequences with those of other avian influenza viruses from public databases, the isolated three tiger-originated-viruses (specially the tig1404 and tig1508 isolate) had a high level of homology with the recently identified HPAI H5N1 viruses, which circulates mainly in chickens and other avians in Yunnan province at the same time^7^ and the southern provinces of China or Southeast Asia, but low levels of homology with isolates from tigers in Shanghai, Jiangsu and Thailand^6^,^21^,^22^. Thanawongnuwech *et al.* previously reported that H5N1 influenza virus transmission occurred between tigers in Thailand in 2004^23^. In the case On August 12^th^, 2015, RNAs from the two died tigers tested positive for the HA and NA gene of the H5N1 virus. The virulence in mice indicated that the tig1508 virus maybe have the ability for transmission and infection in mice through direct contact.

Our results suggested that the *reassortant* different H5N1 virus subclades can successfully cross species barriers from avian to mammal and infect north-east China tigers which might be with the contribution that mutations/substitutions of the gene segments in the tiger originated viruses could enhance virulence or increase the H5N1 virus binding to the α2-6 receptor.

Evolutionary analysis showed that A/tiger /Yunnan /tig1508 /2015(H5N1) including A/peacock/ Yunnan /1 /2015(H5N1) virus and A/peacock/ Yunnan /3 /2015(H5N1) virus which circulates in peacocks and other poultry is a novel reassortant virus originating from H5N1 and H9N2 subtypes influenza A virus. The HI assay demonstrated antiserum from the RE-6 vaccine strain did not inhibit hemagglutination of clade 2.3.2.1c such as tig1508 and peacock isolates, and this change must be considered when evaluating and selecting prepandemic candidate vaccine viruses for the region.

## Materials and Methods

Tissue samples of all deceased tigers, including throat and tracheal swab, lung, liver, spleen, kidney, cardiac with aquae pericardii, and cerebrospinal fluid, were collected to determine the cause of death. Testing for detection of influenza A virus was performed by a reverse transcription PCR method after RNA extraction using a viral RNA kit (Invitrogen, USA)^24^. RNA from the lung, liver, cardiac with aquae pericardii, throat and tracheal swab specimens tested positive for the hemagglutinin gene and neuraminidase gene of the H5N1 virus.

### Virus isolation and gene sequencing

To isolate and characterize the H5N1 viruses, isolates from H5N1 virus RNA positive dead tiger’s lung samples and peacocks’s cloacal swab specimens were injected into 10-day-old specific pathogen free embryonated chicken eggs in a Biosafety Level-3 laboratory. Two peacock isolates (A/peacock/Yunnan /1 /2015(H5N1) and A/peacock /Yunnan /3 /2015(H5N1)) and three tiger originated virus isolates (tig1404, tig1412 and tig1508 isolated from tigers died on April 8^th^, December 15^th^, 2014 and August 12^th^, 2015, respectively.) were chosen for full genome sequencing. The specific RT-PCRs were performed as described previously^25^. The homogenates of the RT-PCR positive samples for tigers, including the lung, liver, cardiac with aquae pericardii, throat and tracheal swab specimens, were centrifuged at lowspeed (6,000 × g) for 10 min at 4 °C, treated with 100,000 U/ml penicillin and 100 μg/ml streptomycin, and either undiluted or 10-fold serially diluted supernatants, and then inoculated into 10-day-old SPF embryonated chicken eggs. Viral titers were then calculated using the Reed and Muench method.

### Genetic and phylogenetic analysis

The nucleotide sequences were analyzed using DNAman (version 6.0) and BLAST (http://blast.ncbi.nlm.nih.gov/Blast.cgi). Phylogenetic analyses were performed using the maximum likelihood (ML) method (MEGA, version 6.0)^26^. The N-Glycosylation sites were predicted to examine the sequence context of Asn-Xaa-Ser/Thr sequins by the NetNglyc server 1.0. The full genome sequences of all isolates have been sumbitted to the GenBank database and GenBank accection numbers are KU057261-KU057300.

### Pathogenicity of the tigers originated H5N1 viruses in mice

To further characterize the virulence of these novel H5N1 viruses in mice, groups of five mice under light CO_2_ anesthesia were inoculated intranasally with 10^1^−10^6^ 50% egg lethal dose (ELD_50_) of tested virus in a volume of 50 μl. Meanwhile, a group of five mice were inoculated with an equal volume of PBS as negative control. All the mice were monitored for mortality daily for 10 days. For virus infectivity and replication testing, groups of three mice were lightly anesthetized with CO_2_ and inoculated intranasally with 10^6^ ELD_50_ of tested virus in a volume of 50 μl, every three inoculated mice were euthanized at 2–5 days post-inoculation, and their organs were collected for virus re-isolation and RT-PCR test. To investigate the virus transmission ability, after 24 hours post-inoculation, three naïve mice were placed in direct contact with the inoculated mice. Virus re-isolation was performed in 10-day-old SPF embryonated chicken eggs.

### The hemagglutination inhibition (HI) test

To test the antigenic relationship of three tiger viruses, we examined serologic cross-reactivity between the tiger originated isolates and the diagnostic antigen of the widely used inactivated reassortant vaccine Re-5 and vaccine Re-6 in Yunnan Province (Table 3). A hemagglutination-inhibition test was conducted to test for Re-5 and Re-6 antiserum against tiger’s viruses^27^. Forty serum specimens were collected from healthy chickens were vaccinated twice with RE-5 vaccine strain (collection date = 2012).

### Statistical analysis

The statistical significance of differences between groups was determined using the Student’s t-test (Graphpad prism 5). A P value < 0.05 was considered statistically significant.

### Ethics Statement

The animal experiment was conducted in accordance with the Regulations for the Administration of Affairs Concerning Experimental Animals approved by the State Council of the People’s Republic of China. All procedures using animals were approved by the Animal Care and Use Committees of Centre for Disease Control and Prevention, Chengdu Military Region.

## Additional Information

**How to cite this article**: Hu, T. *et al.* Fatal influenza A (H5N1) virus Infection in zoo-housed Tigers in Yunnan Province, China. *Sci. Rep.* **6**, 25845; doi: 10.1038/srep25845 (2016).

## Figures and Tables

**Table 1:** Levels of nucleotide sequence identity of tiger originated AIV H5N1 in Yunnan China, 2014–2015.

Virus	Gene	Virus with the highest percentage of nucleotide identity	GeneBank accession no.	Identity, %
A/Tiger /Yunnan /tig1404 /2014 (H5N1)	PB2	A/chicken /tonghai/ 302 /2014(H5N1)	KP732544	99.3
	PB1	A/chicken /tonghai/ 302 /2014(H5N1)	KP732545	99.4
	PA	A/chicken /tonghai/ 302 /2014(H5N1)	KP732546	99.7
	HA	A/chicken /tonghai/ 302 /2014(H5N1)	KP732547	99.4
	NP	A/chicken /tonghai/ 302 /2014(H5N1)	KP732548	99.6
	NA	A/chicken /tonghai/ 302 /2014(H5N1)	KP732549	99.7
	M	A/chicken /tonghai/ 302 /2014(H5N1)	KP732550	99.5
	NS	A/chicken /tonghai/ 302 /2014(H5N1)	KP732551	99.0
A/Tiger /Yunnan /tig1412 /2014 (H5N1)	PB2	A/chicken/Vietnam/NCVD-KA423/2013(H5N1)	KP097840	99.7
		A/duck /Nanchang/6631/2013(H5N1)	KP288313	99.0
	PB1	A/chicken/Vietnam/NCVD-KA423/2013(H5N1)	KP288314	99.2
		A/duck /Nanchang/6631/2013(H5N1)	KP097861	99.0
	PA	A/chicken/Vietnam/NCVD-KA423/2013(H5N1)	KP097882	99.4
		A/duck /Nanchang/6631/2013 (H5N1)	KP288315	99.1
	HA	A/chicken/Vietnam/NCVD-KA423/2013(H5N1)	KP097915	99.2
		A/duck /Nanchang/6631/2013 (H5N1)	KP288316	98.7
	NP	A/chicken/Vietnam/NCVD-KA423/2013(H5N1)	KP097938	99.6
		A/duck /Nanchang/6631/2013 (H5N1)	KP288317	99.4
	NA	A/chicken/Vietnam/NCVD-KA423/2013(H5N1)	KP097971	99.6
		A/duck /Nanchang/6631/2013 (H5N1)	KP288318	99.3
	M	A/chicken/Vietnam/NCVD-KA423/2013(H5N1)	KP097994	99.2
		A/duck /Nanchang/6631/2013 (H5N1)	KP288319	99.0
	NS	A/chicken/Vietnam/NCVD-KA423/2013(H5N1)	KP098015	99.8
		A/duck /Nanchang/6631/2013 (H5N1)	KP288320	99.6
A/Tiger /Yunnan /tig1508/2015 (H5N1)	PB2	A/chicken/Hunan/1/2012(H9N2)	KF714772	98.1
	PB1	A/chicken/Hunan/1/2012(H9N2)	KF714773	98.2
	PA	A/duck/Hunan/S4150/2011(H5N1)	CY146691	98.2
	HA	A/duck/Hunan/S4150/2011(H5N1)	CY146692	97.6
	NP	A/duck/Hunan/S4150/2011(H5N1)	CY146693	98.2
	NA	A/duck/Hunan/S4150/2011(H5N1)	CY146694	97.1
	M	A/chicken/Hebei/FL/2011(H9N2)	KC821206	98.4
		A/chicken/Hunan/1/2012(H9N2)	KF714778	98.1
	NS	A/duck/Hunan/S4150/2011(H5N1)	CY146696	98.8

^*^PB2, basic polymerase 2; PB1, basic polymerase 1; PA, acidic polymerase; HA, hemagglutinin; NP, nucleoprotein; NA, neuraminidase; M, matrix; NS, nonstructural protein.

**Table 2:** Analysis of molecular features associated with AIV virulence, transmissibility, and antiviral resistance in H5N1 AIV isolated from tigers in Yunnan China, 2014–2015.

Protein	Molecular feature or amino acid substitution	Phenotypic effect	tig1404	ig1412	tig1508
HA	Asp94Asn	Increased virus binding to α2-6; enhanced virus fusion	N(Asn)	S(Ser)	N(Asn)
	Ser133Ala	Increased psuedovirus binding to α2-6	A(Ala)	A(Ala)	A(Ala)
	Ser155Asn	Increased virus binding to α2-6	D(Asp)	D(Asp)	D(Asp)
	Thr156Ala	Increased virus binding to α2-6 and increased transmission in guinea pigs	T(Thr)	A(Ala)	T(Thr)
	Asp183Gly	Increased virus binding to α2-6	N(Asn)	D(Asp)	D(Asp)
	Thr188Ile	Increased psuedovirus binding to α2-6	T(Thr)	I(Ile)	I(Ile)
	Lys189Arg	Increased virus binding to α2-6	N(Asn)	R(Arg)	T(Thr)
	Gln192Arg, Gln192His	Increased virus binding to α2-6	K(Lys)	K(Lys)	Q(Gln)
	Lys218Glu	Altered pathogenicity and tissue tropism in mice, emerged in the course of virus replication in a patient	Q(Gln)	R(Arg)	K(Lys)
	Gln222Leu	Increased virus binding to α2-6	Q(Gln)	Q(Gln)	Q(Gln)
	Ser223Asn	Increased virus binding to α2-6, emerged in the course of virus replication in a patient (fatal case)	R(Arg)	R(Arg)	S(Ser)
	Gly224Ser	Increased virus binding to α2-6	G(Gly)	G(Gly)	G(Gly)
	323 to 330 (R-X-R/K-R)	Polybasic cleavage motif sequence required for high pathogenicity of H5N1 avian influenza viruses	RERRR*KR	IERRRRKR	RERRR*KR
NA	49–68 deletion	Enhanced virulence in mice	deletion	deletion	deletion
	Gln116Leu/Lys/Arg (136 in N2)	Reduced susceptibility to zanamivir and oseltamivir	Q(Gln)	Q(Gln)	H(His)
	His254Tyr/Arg (274 in N2)	Reduced susceptibility to oseltamivir and peramivir	Y(Tyr)	Y(Tyr)	Y(Tyr)
PB2	Glu627Lys	Increased virulence in mice	V(Val)	E(Glu)	E(Glu)
	Asp701Asn	Mammalian host adaptation Enhanced replication efficiency increased virulence and transmission in guinea pigs	D(Asp)	D(Asp)	N(Asn)
M1	Asn30Asp	Increased virulence in mice	D(Asp)	D(Asp)	D(Asp)
	Thr215Ala	Increased virulence in mice	A(Ala)	A(Ala)	A(Ala)
M2	Val27Ala	Reduced susceptibility to amantadine and rimantadine	V(Val)	V(Val)	G(Gly)
	Ser31Asn/Gly	Reduced susceptibility to amantadine and rimantadine	S(Ser)	S(Ser)	N(Asn)
NS1	Pro42Ser	Increased virulence in mice	S (Ser)	S (Ser)	S (Ser)
	80–84 deletion	Increased virulence in mice	deletion	deletion	deletion
	Asp87Glu	Increased virulence in mice	E(Glu)	E(Glu)	D(Asp)
	Leu98Phe	Increased virulence in mice	F(Phe)	F(Phe)	F(Phe)
	Ile101Met	Increased virulence in mice	M(Met)	M(Met)	M(Met)
	222–225 (PDZ ligand domain)	Increased virulence in mice	ESEV	ESEV	ESEV

PB2, basic polymerase 2; PB1, basic polymerase 1; PA, acidic polymerase; HA, hemagglutinin; NP, nucleoprotein; NA, neuraminidase; M, matrix; NS, nonstructural protein.

**Table 3:** Results of HI assays using Re-5 antiserum and Re-6 antiserum for avian influenza A(H5N1) isolated from tigers in Yunnan China, 2014–2015*.

Isolate	Isolation date	HI titer ± SD, log2	
		Re-5 antiserum	Re-6 antiserum
A/tiger/Yunnan/Tig1404/2014	2014 Apr	4.825 ± 1.083	4.475 ± 1.753
A/tiger/Yunnan/Tig1412/2014	2014 Dec	4.289 ± 1.160	6.75 ± 0.840
A/tiger/Yunnan/Tig1508/2015	2015 Aug	4.232 ± 1.212	3.675 ± 1.163
A/peacock/Yunnan/1/2015	2015 Jun	4.116 ± 1.141	3.525 ± 1.339
Re-6 diagnostic antigen†	NA	4.245 ± 1.791	8.875 ± 1.090
Re-5 diagnostic antigen†	NA	7.250 ± 0.909	5. 375 ± 1.330

^*^Re-5 (n = 38) and Re-6 (n = 40) antiserum were generated by vaccinating specific-pathogen free chickens with the commercial Re-5 and Re-6 vaccine (Harbin Weike biologic Technology Development Company, Harbin, China). HI titers against the homologous antigen/virus are shown in boldface. HI, hemagglutination inhibition; NA, not applicable. The titre differences were statistically significant by One Way ANOVA (P < 0.05).

^†^The commercial Re-5 and Re-6 diagnostic antigen (including positive and negative control serum) are from Harbin Weike biologic Technology Development Company, Harbin, China.
